# Biochemical Properties of Highly Neuroinvasive Prion Strains

**DOI:** 10.1371/journal.ppat.1002522

**Published:** 2012-02-02

**Authors:** Cyrus Bett, Shivanjali Joshi-Barr, Melanie Lucero, Margarita Trejo, Pawel Liberski, Jeffery W. Kelly, Eliezer Masliah, Christina J. Sigurdson

**Affiliations:** 1 Departments of Pathology and Medicine, University of California, San Diego, La Jolla, California, United States of America; 2 Department of Neuroscience, University of California, San Diego, La Jolla, California, United States of America; 3 Department of Molecular Pathology and Neuropathology, Medical University, Lodz, Poland; 4 Department of Chemistry, The Scripps Research Institute, La Jolla, California, United States of America; 5 Department of Pathology, Microbiology, and Immunology, University of California, Davis, California, United States of America; University of Edinburgh, United Kingdom

## Abstract

Infectious prions propagate from peripheral entry sites into the central nervous system (CNS), where they cause progressive neurodegeneration that ultimately leads to death. Yet the pathogenesis of prion disease can vary dramatically depending on the strain, or conformational variant of the aberrantly folded and aggregated protein, PrP^Sc^. Although most prion strains invade the CNS, some prion strains cannot gain entry and do not cause clinical signs of disease. The conformational basis for this remarkable variation in the pathogenesis among strains is unclear. Using mouse-adapted prion strains, here we show that highly neuroinvasive prion strains primarily form diffuse aggregates in brain and are noncongophilic, conformationally unstable in denaturing conditions, and lead to rapidly lethal disease. These neuroinvasive strains efficiently generate PrP^Sc^ over short incubation periods. In contrast, the weakly neuroinvasive prion strains form large fibrillary plaques and are stable, congophilic, and inefficiently generate PrP^Sc^ over long incubation periods. Overall, these results indicate that the most neuroinvasive prion strains are also the least stable, and support the concept that the efficient replication and unstable nature of the most rapidly converting prions may be a feature linked to their efficient spread into the CNS.

## Author Summary

Prion diseases are fatal neurodegenerative disorders that are also infectious. Prions are composed of a misfolded, aggregated form of a normal cellular protein that is highly expressed in neurons. Prion- infected individuals show variability in the clinical signs and brain regions that selectively accumulate prions, even within the same species expressing the same prion protein sequence. The basis of these divergent disease phenotypes is unclear, but is thought to be due to different conformations of the misfolded prion protein, known as strains. Here we characterized the neuropathology and biochemical properties of prion strains that efficiently or poorly invade the CNS from their peripheral entry site. We show that prion strains that efficiently invade the CNS also cause a rapidly terminal disease after an intracerebral exposure. These rapidly lethal strains were unstable when exposed to denaturants or high temperatures, and efficiently accumulated misfolded prion protein over a short incubation period *in vivo*. Our findings indicate that the most invasive, rapidly spreading strains are also the least conformationally stable.

## Introduction

Prion diseases are fatal neurodegenerative disorders that include Creutzfeldt-Jakob disease (CJD) in humans, bovine spongiform encephalopathy (BSE) in cattle, and chronic wasting disease (CWD) in cervids (reviewed in [1]). These disorders are caused by misfolding of the cellular prion protein, PrP^C^, into a β-sheet-rich, aggregated isoform known as PrP^Sc^ [2]–[5]. PrP^Sc^ can exist as distinct conformational variants or strains, which show strikingly different disease phenotypes even when PrP^C^ sequences are identical [6]–[10]. Prions strains may vary in their aggregate size, stability in chaotropes, PrP epitopes exposed, glycosylation profile, and core of shielded hydrogen atoms as assessed by H/D exchange and mass spectrometry [11]–[15]. Nonetheless, the critical conformational features of PrP^Sc^ that drive rapidly lethal disease remain unclear.

Conformational determinants of PrP^Sc^ that impact the key events in prion pathogenesis are emerging. Incubation periods from time of exposure to terminal disease vary widely among prion strains, sometimes by more than two-fold [16]. Prion stability, or resistance to denaturation, has been assessed in chaotrope-based assays and has revealed that short incubation period strains correlated with unstable PrP^Sc^ in mouse [17], yet with stable PrP^Sc^ in hamster [18]. Together these findings suggest that other factors such as differences in prion structure or cellular processing influence survival times.

PrP^Sc^ particle size varies among prion aggregates, from oligomers to long fibrils, with the most highly infectious PrP^Sc^ size identified as small oligomers [19], [20]. Prion strains also show differences in the amount of proteinase K (PK)-sensitive versus PK-resistant aggregates [21], [22], with some of the most virulent strains having an estimated 80% of aggregates being PK-sensitive [13], [21], [23]. Additionally, the electrophoretic mobility of the PK-resistant core can differ between strains [11]. Thus, the conformational variability among distinct prion strains is pronounced and correlates with some features of disease.

Following entry of prions, many prion strains accumulate in lymphoid tissue during the early stages of disease. Prions spread to the CNS through peripheral nerves in a process known as neuroinvasion [24], [25]. Although it has not yet been directly demonstrated how PrP^Sc^ transits via peripheral nerves, a wealth of indirect evidence exists that prion transport by nerves is a major entry route into the brain [26]–[30]. Manipulating the density of nerves or the distance between nerves and the PrP^Sc^ peripheral reservoirs in lymphoid tissue has a robust impact on neuroinvasion [26], [27], [31]. Additionally, gastric, ocular, or lingual exposure to prions induces prion replication initially at CNS sites with direct neural connections to the entry site, also suggestive of neural transport of prions into the CNS [30], [32]–[34]. Yet little is known about how strains differ in their capacity for neuroinvasion.

Neuroinvasion is dependent upon (1) host and (2) the specific strain. Certain critical host factors that enhance neuroinvasion, such as CD21/35 complement receptor or C1q, have been identified through prion infection studies in which receptors or complement were depleted [35]–[37]. Yet within the same host species, distinct strains show varying abilities to invade the CNS. For example, the mouse-adapted sheep scrapie strain 87 V is poorly neuroinvasive whereas strain 22A is highly neuroinvasive [38]. Yet, the structural determinants that underlie strain differences in CNS entry are unknown. Here we investigated the biochemical properties of prion strains that efficiently or poorly invade the CNS. We demonstrate that the most rapidly lethal strains are highly neuroinvasive, physically unstable, and form primarily diffuse aggregates in the brain.

## Results

### The capacity of prions to neuroinvade is strain dependent

**Figure 1:**
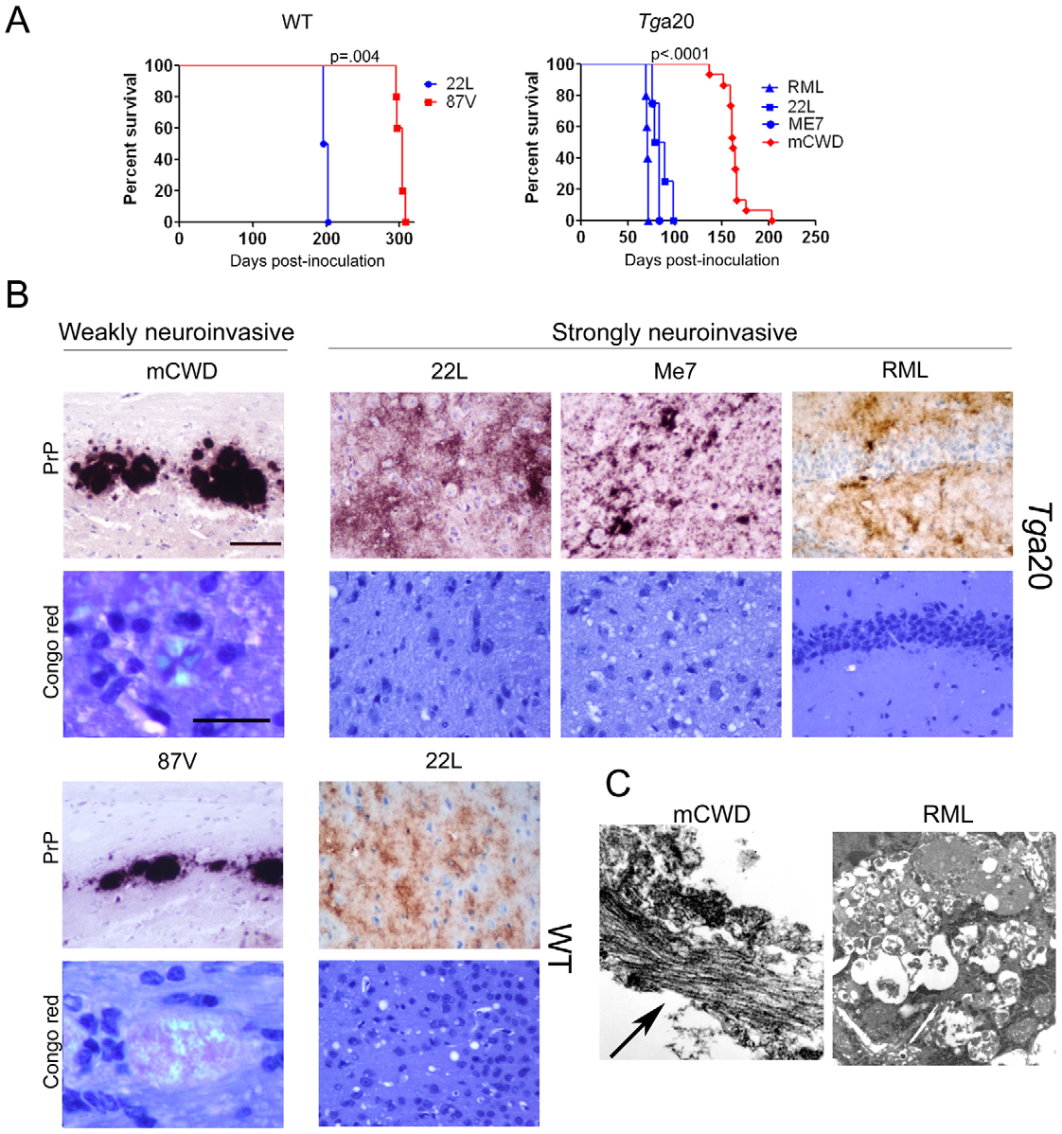
Characterization of prion phenotypes in mice infected with different prion strains. (A) Survival curves of *Tg*a20 and WT mice IC inoculated with prions indicate that strains induced terminal clinical disease after short (blue) or long (red) incubation periods. P value is derived from a log-rank test. (B) Representative brain sections immunolabelled for PrP show the typical large, dense plaques of mCWD and 87 V and the diffuse patchy aggregates and small plaques of 22 L, ME7, and RML. Only the mCWD and 87 V plaques stained with Congo red. Scale bars = 100 µm (PrP) and 50 µm (Congo red). (C) Ultrastructure of the plaques from mCWD and RML show long fibrils in the mCWD-infected brain (arrow). No fibrils were seen in the RML-infected brain, although dead cells were observed.

**Figure 2:**
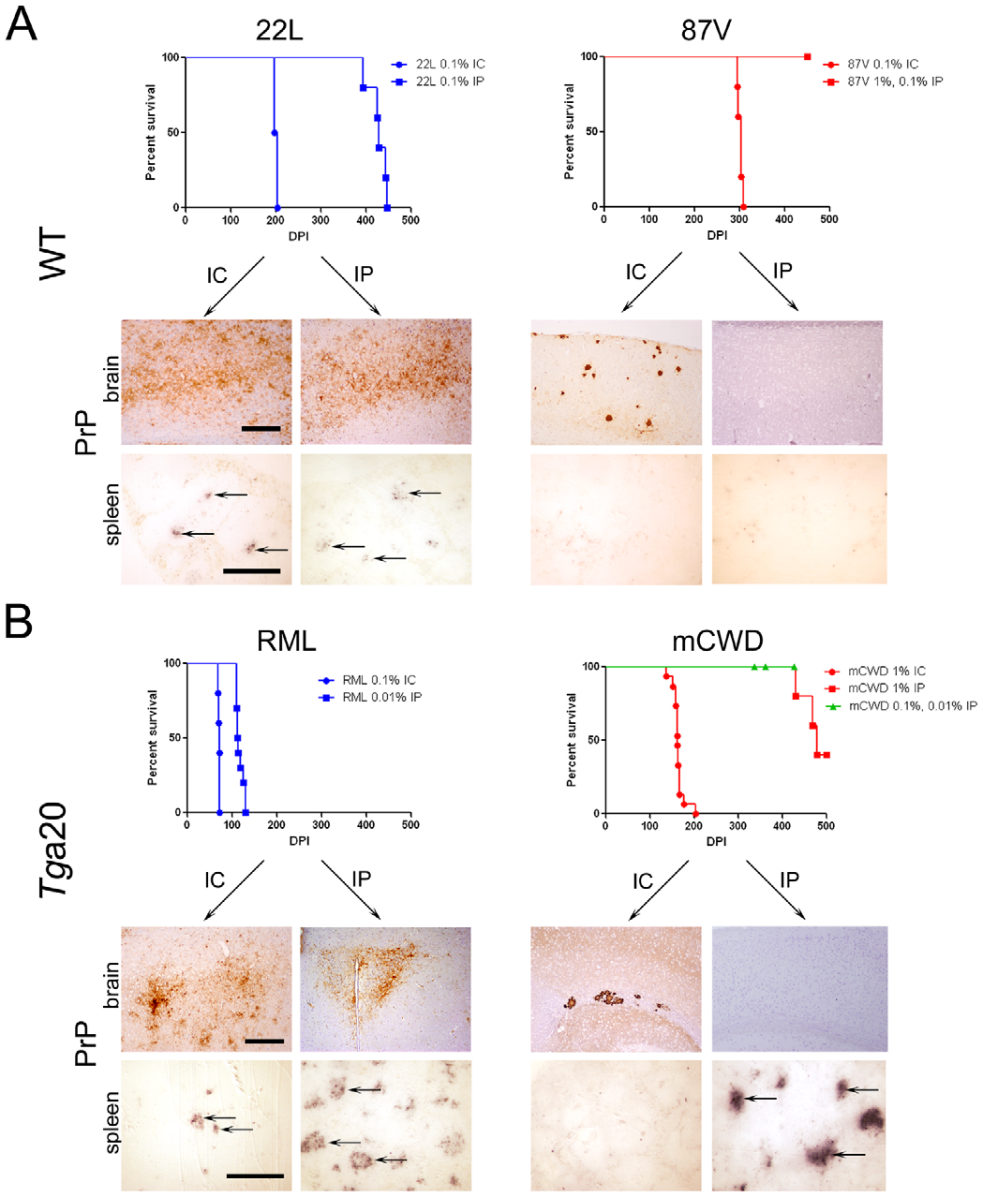
Comparison of mice inoculated with prion strains by intracerebral (IC) and intraperitoneal (IP) routes. (A) WT mice. Survival curves and PrP^Sc^-immunolabelled brain sections indicate that 22 L prions cause terminal disease after either IC or IP exposure, whereas 87 V prions cause terminal disease only after IC exposure. Histoblots of spleen show PrP^Sc^ in the lymphoid follicles of 22 L, but not 87 V-inoculated mice. (B) *Tg*a20 mice. Survival curves and PrP^Sc^-immunolabelled brain sections similarly show that RML prions cause terminal disease after either IC or IP exposure. mCWD prions cause terminal disease after IC and in some mice after IP exposure, suggesting inefficient neuroinvasion. Histoblots of spleen show PrP^Sc^ in the lymphoid follicles in RML-, and some mCWD-inoculated mice. Scale bar = 400 µm (brain) or 1 µm (spleen).

To compare the pathogenesis of prion strains, we inoculated WT mice (VM/Dk background) with mouse-adapted strains 22 L or 87 V by intracerebral (IC) or intraperitoneal (IP) routes. Prion infection in brain was determined by three independent assays: western blot, histoblots, and immunohistochemistry. Following IC inoculation, both 22 L and 87 V led to terminal prion disease in all mice, although with a significantly longer incubation period in 87 V-exposed mice (22 L: 200±2 days; 87 V: 302±2 days) (Figure 1A). Yet after IP inoculation, only 22 L was neuroinvasive and led to the consistent development of clinical prion disease and PrP^Sc^ in the brain (5/5, 100% attack rate, Figure 2A, left panel). No prions were detected in the brains of mice inoculated IP with strain 87 V (0/6), consistent with previous reports indicating that CNS invasion was inefficient for this strain (Figure 2A, right panel).

To determine how additional strains vary in their ability to neuroinvade, we inoculated mice expressing WT mouse PrP^C^ (*Tg*a20 mice) with mouse-adapted CWD (mCWD) and mouse-adapted scrapie strains RML, 22 L, and ME7 by the IC route. *Tg*a20 mice overexpress a WT PrP sequence that varies from VM/DK mice by two dimorphisms (108L/F and 189T/V) [39]. Here we again found that following IC inoculation, the strains led to disease with 100% attack rate but showed significant differences in the time to terminal prion disease. The mean incubation period for strains RML, 22 L, and ME7 was 71–85 days, whereas the mean incubation period for strain mCWD was significantly longer at 164 days (Figure 1A). Upon IP inoculation of RML and mCWD, the rapid strain RML developed prion disease with an incubation period that was extended 40 days beyond that of the IC route and had an attack rate of 100% (n = 10). In contrast, the slow mCWD strain developed prion disease with an incubation period of approximately 460 days and a very low attack rate of 20% (3/15) (Figure 2B). ME7 and 22 L are well-established to be strongly neuroinvasive strains following IP inoculation [35], [40]–[43]. Taken together with the WT mouse infections, we found that the rapid strains (22 L, RML) tended to be strongly neuroinvasive with all mice developing prion disease following a peripheral exposure, whereas the slower strains (87 V, mCWD) tended to be weakly or non-neuroinvasive.

### Morphologic properties of strongly and weakly neuroinvasive (NI) prions

We found dramatic differences in the PrP^Sc^ aggregate morphology in brains of mice that were IC inoculated with strongly and weakly NI strains. In the WT brain, the strongly NI strain, 22 L, led to diffuse PrP^Sc^ deposits that did not stain with the amyloid binding dye Congo red, hence deposits are referred to as noncongophilic. In contrast, the weakly NI strain, 87 V, led to dense, large aggregates that bound CR, and are referred to as congophilic (Figure 1B). Similarly, in the *Tg*a20 brain, RML, ME7, 22 L, all strongly NI strains [31], [35], [40]–[43], led to diffuse, fine 2–5 µm aggregates and occasionally small 10–15 µm plaques that were noncongophilic, whereas the weakly NI strain, mCWD, led to focal, dense, large 20–50 µm plaques that were congophilic (Figure 1B). Ultrastructurally, the strongly NI strains had no visible fibrils consistent with previous reports for ME7 and RML [44], [45], whereas the weakly NI prion mCWD consistently had long fibrils visible within the plaques (Figure 1C and Figure S1). Plaques of fibrils have been previously observed for 87 V [45].

We then compared the prion aggregate morphology of the same strain entering the brain following either an IC or IP exposure. The strongly NI strains showed identical aggregate morphologies in the brains of mice exposed either by the IC or IP routes (Figure 2A,B). However in the weakly NI strains, there were surprising differences in the PrP^Sc^ aggregate morphology. After IP inoculation, the weakly NI strains showed none of the large congophilic aggregates in the brain that were seen after the IC inoculation, although scattered diffuse aggregates were seen in the brain of three *Tg*a20 mice IP exposed to mCWD (data not shown).

### Peripheral prion replication varies among the strongly and weakly NI prion strains

We next assessed whether PrP^Sc^ accrued in the lymphoid tissues after IP inoculation with the different strains. Here we found that PrP^Sc^ was detectable in spleens of all mice inoculated with the strongly NI strains RML and 22 L, but in none of the weakly NI 87 V-inoculated and only approximately 20% of mCWD-inoculated mice (Figure 2). Thus, the strongly NI strains also developed a more robust accumulation of prions in the lymphoid tissues.

### Electrophoretic mobility and glycoform profiles of the strains

Having found that the less neuroinvasive prions form large, dense congophilic core plaques in histologic sections suggested that there were structural differences among the strains that may alter their ability to spread to the brain. To test this hypothesis, we first investigated the structural and biochemical differences underlying the biological properties of the strongly and weakly NI prions. We compared the mobility of the PK resistant core fragment and the ratios of di-, mono- and unglycosylated PrP^Sc^ glycoforms. In assessing the PK-resistant core fragment, we found no differences in the electrophoretic mobility among the strains. The glycoform ratios varied among the strains, however neither the PK-resistant PrP core size nor the glycoform profile correlated with neuroinvasion ability (data not shown).

### Strongly NI strains degrade with limited proteolysis at low chaotrope concentrations

**Figure 3:**
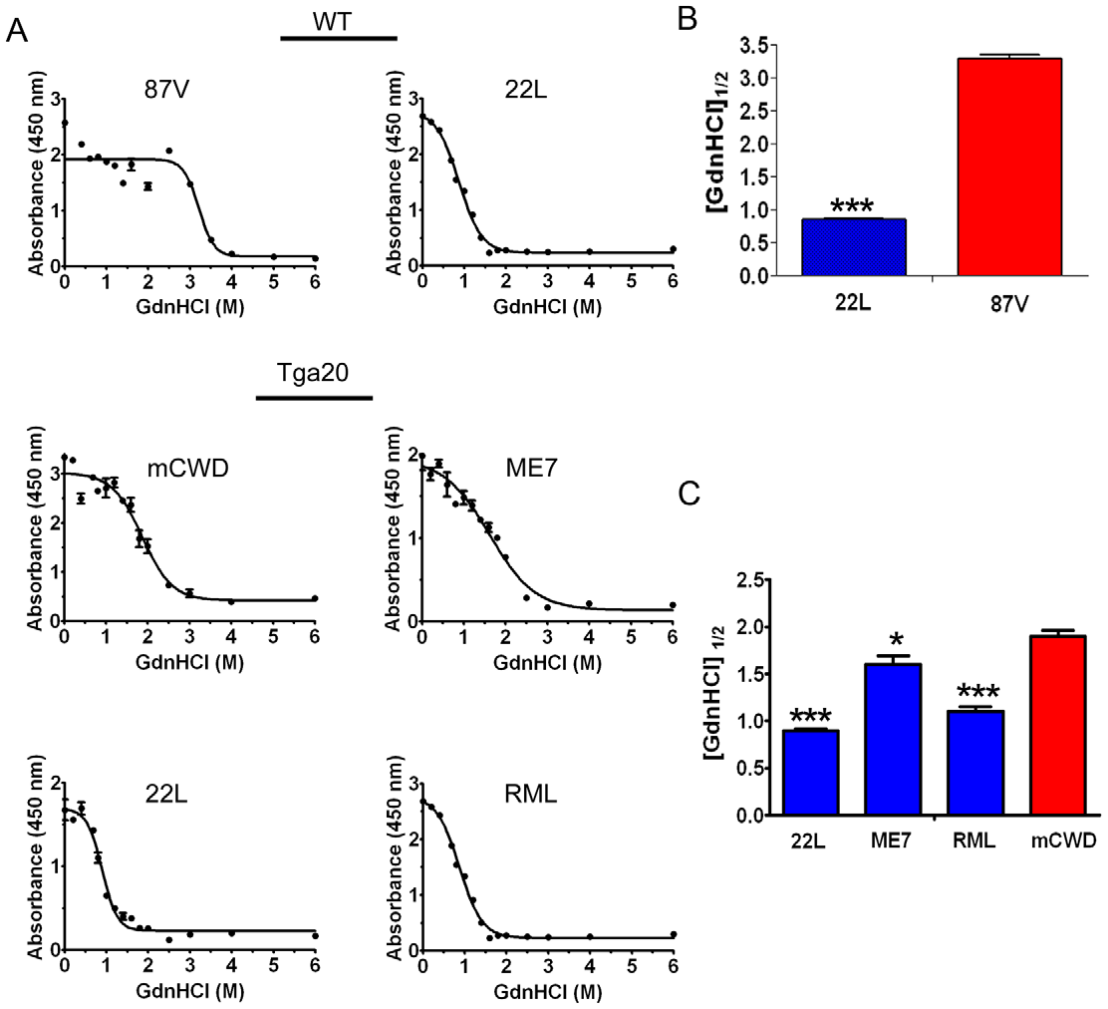
Conformational stability of prion strains. (A) Brain homogenate was treated with increasing concentrations of GdnHCl and digested with PK, and PrP^Sc^ was detected by ELISA. The denaturation curves were plotted as PrP^Sc^ absorbance signals against the GdnHCl concentration and fitted to a sigmoidal function. The half-maximal denaturation occurred at GdnHCl concentrations that were greater for the weakly neuroinvasive strains (mCWD and 87 V) than the strongly neuroinvasive strains (22 L, ME7, RML). (B) Plot of [GdnHCl]_1/2_ values for WT mice infected with 87 V and 22 L. (C) Plot of [GdnHCl]_1/2_ values for *Tg*a20 mice inoculated with 22 L, ME7, RML and mCWD. Error bars are present for all data points. * and *** indicate P values of <0.01 and .0001, respectively when comparing the indicated strain with mCWD.

Prion strains have been shown to vary dramatically in their conformational stability. To next determine how the strongly and weakly NI prions varied in their stability in chaotropes, we exposed brain homogenates to 14 concentrations of guanidine hydrochloride (GdnHCl) ranging from 0 M to 6 M. We then diluted the samples to 0.15 M GdnHCl, digested with PK, denatured and measured the PrP^Sc^ remaining by ELISA, and calculated the [GdnHCl]_1/2_. ELISA was used to quantify PrP^Sc^ since western blots of murine prion strains showed no visible mobility shift in PK-resistant PrP^Sc^ at the higher GdnHCl concentrations (data not shown). This suggests global PrP^Sc^ denaturation and complete PrP digestion with PK occurs, consistent with the results of Peretz and colleagues [46]. Interestingly, the strongly NI, nonfibrillar strains showed the lowest [GdnHCl]_1/2_, which was 0.9±0.2, 1.1±0.1 and 1.6±0.1 for the 22 L, RML and ME7 strains, respectively. The [GdnHCl]_1/2_ was significantly higher for the weakly neuroinvasive strains mCWD at 1.9±0.1 and 87 V at 3.2±0.1. (Figure 3A–C). These results indicate that the strongly NI, nonfibrillar, noncongophilic strains 22 L, RML, and ME7 were less stable in chaotropes as compared to the weakly NI, fibrillar, congophilic strains mCWD and 87 V.

### Strongly NI strains readily disassemble at low temperatures

**Figure 4:**
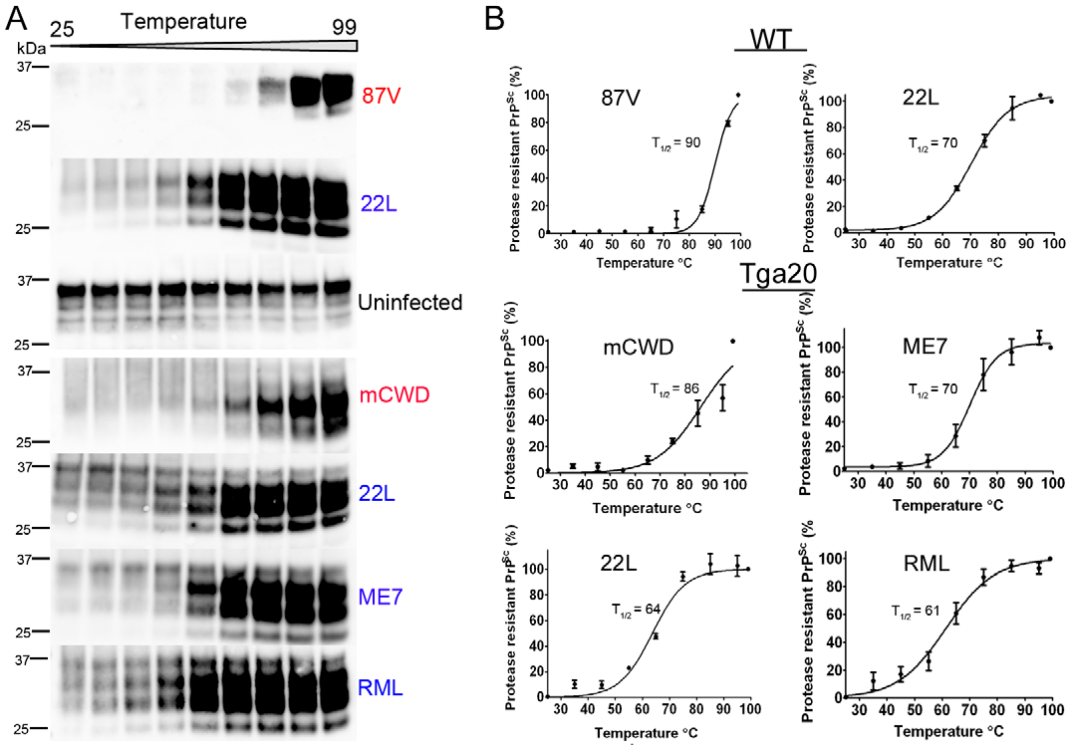
Thermal stability of strongly and weakly neuroinvasive prion strains. (A) PK-digested brain samples were subjected to increasing temperatures followed by SDS-PAGE. (B) Monomers were quantified by band intensity analysis, plotted against temperature, and fitted to a sigmoidal function. The PrP signal measured at 99°C was considered as total PrP^Sc^ (100%). The temperature required for 50% PrP^Sc^ disassociation into monomers is lower for the more neuroinvasive prion strains (22 L, ME7, RML). A faint band at 37°C may represent residual PrP^C^ and was not included in the signal quantification. Values represent mean ± standard error.

To further confirm the stability differences among strains, we also assessed the thermostability of the prion aggregates. Here we digested brain homogenate with PK and exposed PrP^Sc^ to a thermal gradient from 25–99°C in the presence of 1.6% SDS for 6 minutes, then performed one dimensional denaturing gel electrophoresis and quantified levels of monomeric PrP, as has been previously shown with yeast prions [47]. These measurements indicate how readily the aggregates disassemble into monomers at increasing temperatures. We determined the temperature at which half of the total PrP^Sc^, measured at 99°C, appeared as monomers (T_1/2_). The thermostability and chaotrope stability measurements showed remarkable agreement. Whereas the strongly NI strains showed T_1/2_ from 61–70°C, the weakly NI, fibrillar strains disassembled at higher temperatures, with a T_1/2_ from 86–90°C (Figure 4). These results again indicated that the strongly NI, noncongophilic strains formed less stable aggregates.

### Strongly neuroinvasive prion strain has high levels of insoluble PrP^Sc^

**Figure 5:**
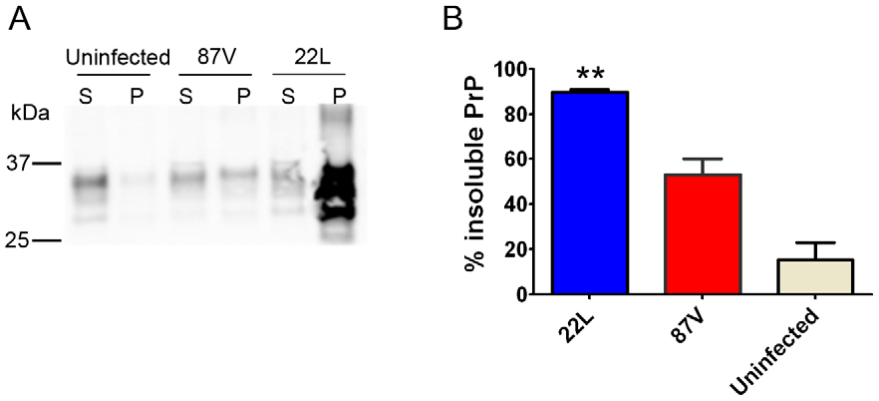
Soluble to insoluble PrP ratio varies depending on the prion strain. (A) Brain homogenate was ultracentrifuged, and the supernatant and pellet fractions were subjected to SDS-PAGE and immunoblotting for PrP. The strongly neuroinvasive strain 22 L has significantly higher levels of insoluble PrP as compared to the weakly neuroinvasive 87 V. (B) Plot of the percentage of insoluble PrP. S = supernatant, P = pellet. ** indicates a P value of <0.001 for 22 L as compared with 87 V.

To assess the relative efficiencies of PrP conversion, we measured the amount of soluble and insoluble PrP in brain at terminal disease for each WT strain. The insoluble PrP fraction was significantly higher in the strongly NI 22 L versus the weakly NI 87 V, at 90%±1 versus 53%±7 insoluble PrP, respectively (Figure 5). The 22 L strain notably accumulated 11±4-fold more total PrP^Sc^ at terminal disease than 87 V, which occurred over a shorter incubation period. These data suggest that the noncongophilic 22 L is more efficient at rapidly converting PrP^C^ *in vivo* as compared to the congophilic 87 V strain. The *Tg*a20 strains showed highly variable insoluble∶soluble PrP ratios, likely due to the high PrP^C^ expression.

Since the relative level of PK-sensitive PrP^Sc^ may influence the neuroinvasive ability of a strain, we quantified the total insoluble and PK-resistant PrP^Sc^ of strains 22 L and 87 V. PK-digested and non-digested aliquots of brain homogenate were ultracentrifuged, and PrP^Sc^ was measured in the pellet fractions. Interestingly the levels of PK-resistant PrP^Sc^ were similar and consisted of 40±3% and 46±6% of the total insoluble PrP for 22 L and 87 V, respectively, indicating that >50% of the total insoluble PrP^Sc^ was PK-sensitive (Figure S2).

To determine whether the total insoluble PrP^Sc^ more readily dissembled into monomers at lower temperatures as compared to the PK-resistant fraction, the thermostability measurements were repeated on total insoluble PrP^Sc^ in the absence of PK. The T_1/2_ was similar to the values determined for the PK-resistant PrP^Sc^ for both the 22 L and 87 V (T_1/2_: 22 L = 65°C±5; 87 V = 92°C±3) (Figure S3).

## Discussion

Infectious prions invade the body through peripheral sites such as the gastrointestinal tract, and following amplification in lymphoid tissues for some strains, spread mainly via peripheral nerves into the CNS [48]. Experimentally, certain fibril-forming prion strains replicate peripherally and do not enter the CNS, leading to a persistence of prions in extraneural tissues [24], [38], [49]. GPI-anchorless prion strains are also fibrillar and also show infrequent neuroinvasion after inoculation by tongue, ocular, intravenous, or intraperitoneal routes [50]. Natural infection with similar weakly or non-neuroinvasive strains may yield asymptomatic, long-term carriers of infectious prions, thus could pose a risk for transmission to other humans or animals. Here we investigated the pathologic phenotype and the biochemical properties of strongly and weakly neuroinvasive prion strains using a range of assays. Our results establish that the strongly neuroinvasive murine strains are less stable and efficiently accumulate PrP^Sc^ over a short incubation period.

### The strongly and weakly neuroinvasive prions form distinct aggregate morphologies after intracerebral challenge

The strongly and weakly neuroinvasive mouse strains showed profound differences in the PrP aggregate morphology and incubation period to terminal disease. After intracerebral inoculation, strongly neuroinvasive strains form diffuse, nonfibrillar PrP aggregates and mice rapidly progressed to terminal disease. In contrast, weakly neuroinvasive strains form dense, congophilic, fibrillar plaques and mice slowly progressed to terminal disease. These findings suggest that the congophilic, fibrillar PrP^Sc^ accumulates slowly or is less toxic, the latter consistent with recent evidence that fibrils in general are less toxic than oligomers [51]. Interestingly, there are new reports of strains that form large, dense plaques and show prolonged incubation periods, such as vCJD prions in transgenic mice expressing human 129VV PrP^C^ [52] and Gerstmann–Straussler-Scheinker (GSS) prions in TgPrP101LL mice [53]. Similar to our mCWD strain, the vCJD and GSS mouse strains show a predilection for the corpus callosum region [52], [53].

### PrP^Sc^ spread to the CNS

We found that the pathogenesis diverged among the strains inoculated IP, in that only the diffuse, noncongophilic strains were strongly neuroinvasive. Since several murine prion strains inoculated IP require amplification in the lymphoid tissues for efficient neuroinvasion to occur [54], [55], it is possible that the strongly neuroinvasive prion strains replicate readily to higher levels in lymphoid tissue, which may facilitate spread through peripheral nerves. Here the strongly neuroinvasive strains had more abundant PrP^Sc^ aggregates in the spleen, and had a short incubation period after IC inoculation. Together these findings suggest that the strongly neuroinvasive strains could propagate efficiently in lymphoid tissues, which may enhance neuroinvasion.

What is the biochemical basis for a strain's efficient spread to the CNS? Marked differences were detected in the stability assays where the strongly neuroinvasive strains were the most unstable. The stability could account for the differences in the disease pathogenesis. First, it is possible that the unstable strains have small PrP^Sc^ aggregates that efficiently spread via peripheral nerves to the CNS. Consistent with this hypothesis, previous studies have shown that small or low density PrP aggregates were unstable and rapidly lethal after IC inoculation [20]. Whether these strains also spread efficiently after IP inoculation is unknown. Second, the higher stability of the congophilic strains may lead to such slow fragmentation and PrP^C^ recruitment that incubation times exceeding the mouse lifespan would be required for prion replication in spleen and spread to the CNS. Although we did not detect splenic PrP^Sc^ in 87 V-infected mice, others have shown with sensitive infectivity assays that 87 V accumulates in the spleen at early timepoints post-inoculation [38]. This suggests that the failure of 87 V to spread to the CNS was not due to a delayed replication in the spleen [38]. A third possibility is that instability of a strain generates high levels of infectious particles necessary to initiate peripheral nerve spread. Indeed, a report of 87 V indicates that very high doses of infectious prions can lead to brain entry [56]. The lymphoid tissues may serve to amplify PrP^Sc^ to high levels near nerve terminals, and thus efficient lymphoid PrP^Sc^ amplification may be crucial for neuroinvasion after IP inoculation. Alternatively, the strain may change after replication in the spleen. Lastly, it is possible that the instability does not underlie the efficient spread of prions, but that there is another yet undiscovered factor, such as a PrP^Sc^ interacting protein, that is required for spread. These possibilities are not mutually exclusive.

### PK-sensitive PrP^Sc^

Previous studies indicate that for many strains, a major fraction of PrP^Sc^ consists of PK-sensitive conformers [57], consistent with our findings of 87 V and 22 L. In human CJD cases, higher levels of stable PK-sensitive conformers were associated with extended disease duration [58]. Similarly, in hamster strains, high levels of PK-sensitive PrP^Sc^ correlate with longer incubation periods [13]. In mice, the level of PK-sensitive forms was found to be higher in RML as compared to ME7 [59]. Although a full characterization of the role of the PK-sensitive and resistant conformers in neuroinvasion is beyond the scope of this study, it will be interesting in future studies to determine which fraction is more involved in neuroinvasion.

### Congophilic prions

Is congophilia predictive of biological behavior? CR staining has been a gold-standard for the histologic diagnosis of amyloid fibrils, in which tightly interdigitated β-sheets are aligned perpendicular to the fibril axis. Recently, CR was shown to bind parallel to the fibril axis in a surface groove of yeast Het-s fibrils, and to require electrostatic interactions for binding [60]. One key residue exchange in Het-s abrogated the binding of CR, even though fibrils were present, thus CR-negative status of aggregates does not necessarily indicate a lack of fibrillar structure. Nevertheless, the noncongophilic prion strains RML and ME7 used here previously have not revealed fibrils after direct examination of the brain ultrastructure [44], [45]. In studies of partially purified hamster prions, fibrils were found only when PrP^Sc^ was truncated by digestion with PK [61], [62]. To our knowledge, fibrils of full length PrP have not been observed for noncongophilic prion strains [62]. In contrast, fibrils are readily detected for strains mCWD [63] and 87 V [45]. Taken together, these studies suggest that for the mCWD and 87 V strains, congophilia correlates with plaques of PrP fibrils.

### Prion stability in mice and yeast

Notably, the unstable and stable prion strains reported here share properties with the yeast Sup35 prion strains, Sc4 and SCS. Sc4 prions are thermally unstable, readily fragment to generate short fibrils, and have low levels of soluble monomers, whereas the SCS prions are highly thermally stable, rarely fragment, develop long fibrils, and have high levels of soluble monomers [64]. By comparison, strain 22 L, like Sc4, is thermally unstable and has low levels of soluble monomers, whereas 87 V, like SCS, is highly stable with long fibrils and high levels of soluble monomers. Although we did not directly measure fibril breakage or the propensity to shear, in yeast strains, low thermal stability correlates with high fragmentation rate [64]. Whether this is also true for PrP remains to be determined.

Recent studies in yeast have shown that transmission of a prion aggregate to the daughter cell selects for a certain aggregate size [65], which is governed in part by fragmentation properties. In mice and yeast, the prion stability appears to greatly influence prion amplification, and lends support to the proposed mathematical model that fibril breakage is a dominating factor in the kinetics of prion propagation [66].

This study shows that the more unstable, noncongophilic and nonfibrillar murine prion strains efficiently spread from extraneural sites to the central nervous system. Future studies on the relationship between the biochemical properties of misfolded proteins and the disease phenotype are essential for deciphering how aggregates spread in prion and other protein misfolding diseases.

## Materials and Methods

### Ethics statement

All procedures involving animals were performed to minimize suffering and were approved by the Institutional Animal Care and Use Committee at UC San Diego. Protocols were performed in strict accordance with good animal practices, as described in the *Guide for the Use and Care of Laboratory Animals* published by the National Institutes of Health.

### Prion inoculations

WT (VM/Dk inbred mice, kindly provided by Dr. Byron Caughey) or *Tg*a20 transgenic mice [67] (groups of n = 4–15 mice) were intracerebrally inoculated into the left parietal cortex with 30 µl of a 0.1% or 1% (w/v) prion-infected brain homogenate prepared from terminally diseased mice. Strain 87 V was a gift from Dr. Thomas Wisniewski. Uninfected brain homogenate served as a negative control. Intraperitoneal inoculations were performed using 100 µl of a 0.1% or 1% prion-infected or uninfected brain homogenate. Mice were monitored three times weekly, and TSE was diagnosed according to clinical criteria including ataxia, kyphosis, stiff tail, hind leg clasp, and hind leg paresis. Mice were sacrificed at the onset of terminal disease and incubation period was calculated from the day of inoculation to the day of terminal clinical disease. Mice were maintained under specific pathogen-free conditions.

### Histoblots

Histoblots were performed on tissue cryosections as reported in Taraboulos et al. [68], using up to 100 µg/ml of PK for the digestion of PrP^C^. Histoblots were developed using the anti-PrP POM1 antibody (epitope in the globular domain, amino acids 121–231, a kind gift from Dr. Adriano Aguzzi) [69].

### Histopathology and immunohistochemical stains

Two-µm thick sections were cut onto positively charged silanized glass slides and stained with hematoxylin and eosin, or immunostained using antibodies for PrP (SAF84) or GFAP for astrocytes. For PrP staining, sections were deparaffinized and incubated for 5 min in 88% formic acid, then washed in water for 5 min, treated with 5 µg/ml of proteinase-K, and washed in water for 5 min. Sections were then autoclaved in citrate buffer (pH 6), cooled for 3 min, and washed in distilled water for 2 min. Immunohistochemical stains were performed using the TSA Plus DNP kit (PerkinElmer). Sections were blocked and incubated with anti-PrP SAF-84 (SPI bio; 1∶400) for 45 min followed by anti-mouse HRP (Jackson Immunolabs; 1∶500) for 30 min. Slides were then incubated with anti-DNP-HRP (PerkinElmer, 1∶100) for 30 min, followed by 6 min incubation with DAB. Sections were counterstained with hematoxylin.

### Western blotting for PrP^Sc^ in brain and spleen

Samples were electrophoresed in 10% Bis-Tris SDS-PAGE gels (Invitrogen), transferred onto a nitrocellulose membrane, and PrP detected using the anti-PrP primary antibody POM1 and an HRP-conjugated anti-mouse IgG secondary antibody. The blots were developed using a chemiluminescent substrate (ECL detection Kit, Pierce) and visualized on a Fuji LAS 4000 imager. Quantification of PrP^Sc^ glycoforms was performed using Multigauge V3 software (Fujifilm). Prior to western blotting, PrP^Sc^ was concentrated from the brain homogenates of IP inoculated mice by sodium phosphotungstic acid precipitation as previously described [70].

### Electron microscopy

Tissues were post-fixed in osmium tetroxide, embedded in epon araldite, sectioned with the ultramicrotome, then collected on grids and post-stained using saturated uranyl acetate solution and bismuth subnitrate. Grids were analyzed with a Zeiss EM10 electron microscope.

### Thermostability assay

Brain homogenate in Tris lysis buffer (10 mM Tris-HCl pH 7.4, 150 mM NaCl, 2% sarcosyl) was digested with 50 µg/ml PK for 30 min at 37°C and then treated with phenylmethylsulfonyl fluoride (PMSF) (2 mM final concentration) and Complete TM protease inhibitor (Roche). Individual aliquots were incubated in 1.6% SDS (final) and subjected to temperatures ranging from 25°C to 99°C in 10° intervals for 6 min with shaking at 1000 rpm. Western blotting was performed on the samples by electrophoresis in a 10% Bis-Tris SDS-PAGE gel as described above. PrP signals from monomers were captured and quantified using a Fujifilm LAS-4000 imager and Multigage software. Each strain was analyzed in at least 3 separate experiments using 3–5 mice.

### Conformation stability assay

Prion strain stability in guanidine hydrochloride (GdnHCl) was performed as previously described [14] with minor modifications. Briefly, brain homogenates in Tris lysis buffer were incubated in GdnHCl for 1 hr and then diluted with lysis buffer to a final concentration of 0.15 M GdnHCl. Samples were digested with PK at a ratio of 1∶500 (1 µg PK : 500 µg total protein) for 1 hr at 37°C, treated with protease inhibitors, and centrifuged at 18,000 g for 1 hr at 4°C. The pellets were washed with 500 µl of 0.1 M NaHCO_3_, pH 9.6 and centrifuged for 20 min at 18,000 g. Pellets were denatured in 6 M GdnSCN for 20 min, diluted 2× with 0.1 M NaHCO_3_ and coated passively onto an ELISA plate. PrP was detected with anti-PrP biotinylated-POM1 antibody and a streptavidin HRP-conjugated anti-mouse IgG secondary antibody. The signals were detected with a chemiluminescent substrate (1-Step Ultra TMB-ELISA, Thermo-Scientific). Each strain was analyzed in at least 3 separate experiments using 3–5 mice. Statistical analysis was performed using a Student's *t* test.

### Quantification of soluble and insoluble PrP

Brain homogenate in Tris lysis buffer was centrifuged at 150,000 g for 1 hr at 4°C. The supernatant and pellet were separately collected. Proteins in the supernatant were precipitated using cold methanol. Supernatant and pellet proteins were then analyzed and quantified by western blotting for PrP. Each strain was analyzed in at least 3 separate experiments using 3–5 mice.

## Supporting Information

Figure S1 Ultrastructure of the mCWD-infected brain shows plaques of long fibrils.
(TIF)

Figure S2 PK-sensitive and PK-resistant PrP^Sc^. (A) PK-digested and undigested brain samples were ultracentrifuged and the insoluble fractions were analyzed by SDS-PAGE and immunoblotting for PrP. The WT sample shows a faint signal in the undigested sample. (B) The PrP signals were quantified and revealed no significant difference in the percentage of PK-resistant PrP in the total insoluble PrP for 22 L and 87 V [n = 4 (22 L); n = 3 (87 V)].
(TIF)

Figure S3 Thermal stability of total insoluble PrP^Sc^ for prion strains 22 L and 87 V. (A) The insoluble fraction of brain homogenate was subjected to temperatures from 25°C to 99°C followed by SDS-PAGE. The temperature required for 50% PrP^Sc^ disassociation into monomers is lower for the more neuroinvasive prion strain 22 L than for 87 V.
(TIF)
