# Supplementary figures and images for: Biochemical Properties of Highly Neuroinvasive Prion Strains
Source: PLoS Pathog. 2012 Feb 2;8(2):e1002522. doi: 10.1371/journal.ppat.1002522 (PMC3271082; doi:10.1371/journal.ppat.1002522)

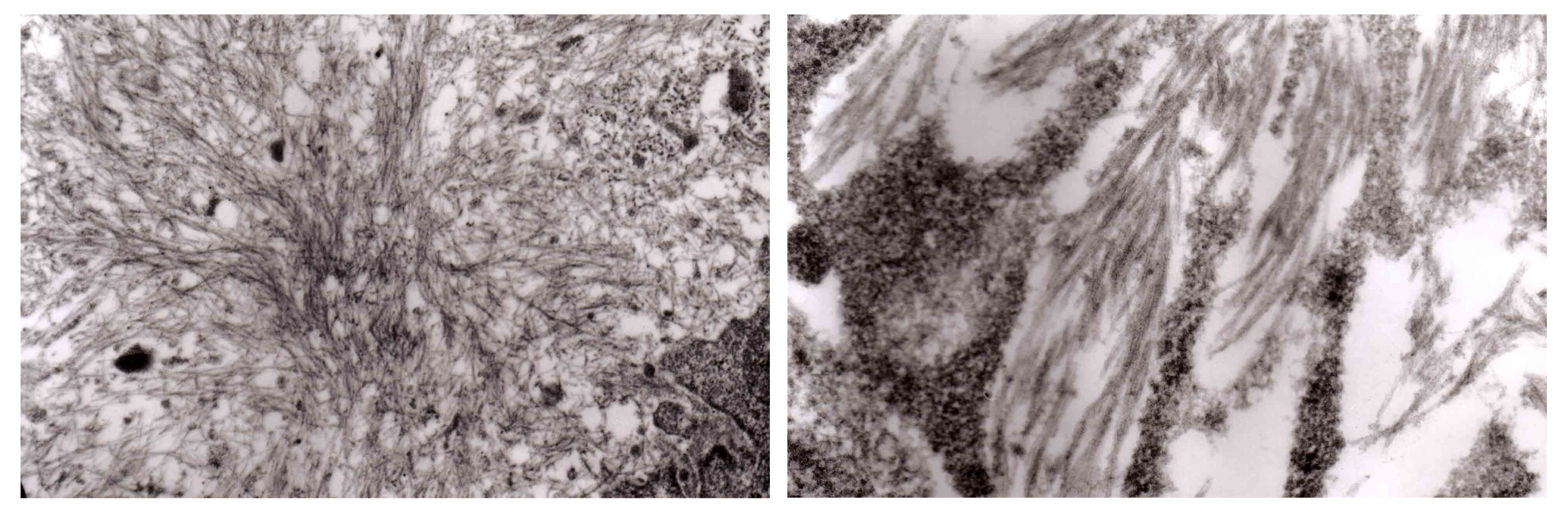

Supplement: Figure S1 — Ultrastructure of the mCWD-infected brain shows plaques of long fibrils. (TIF) [file ppat.1002522.s001.tif]

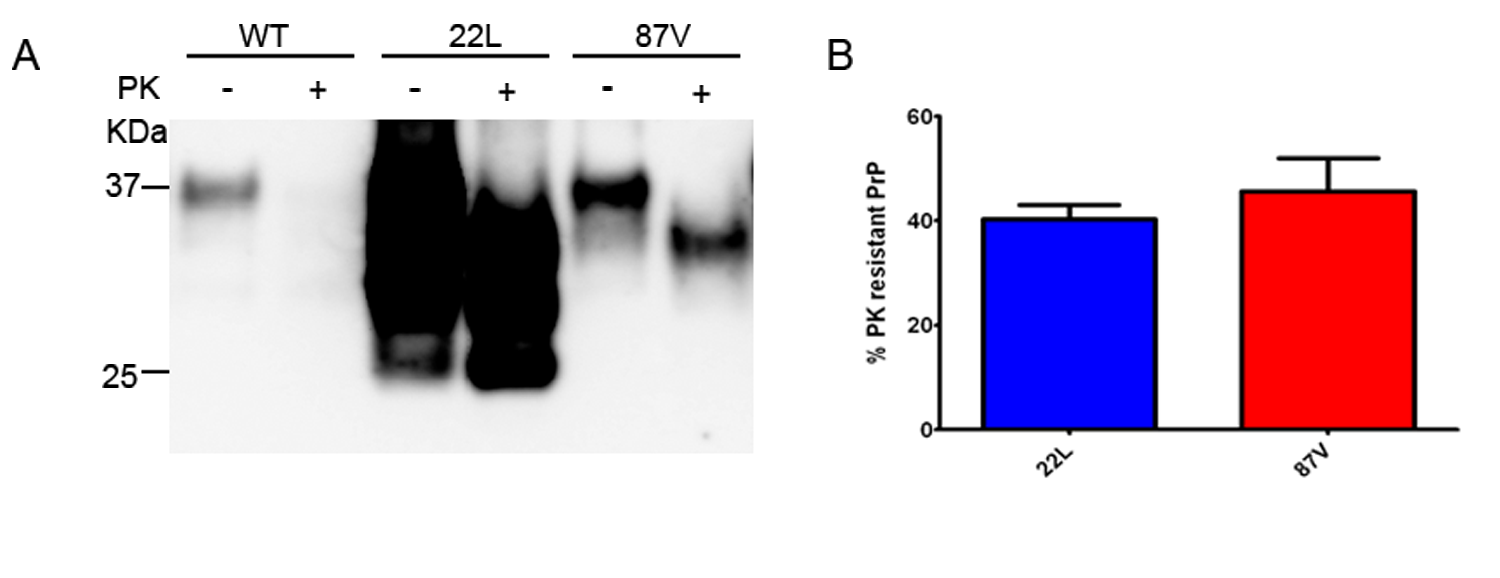

Supplement: Figure S2 — PK-sensitive and PK-resistant PrPSc. (A) PK-digested and undigested brain samples were ultracentrifuged and the insoluble fractions were analyzed by SDS-PAGE and immunoblotting for PrP. The WT sample shows a faint signal in the undigested sample. (B) The PrP signals were quantified and revealed no significant difference in the percentage of PK-resistant PrP in the total insoluble PrP for 22 L and 87 V [n = 4 (22 L); n = 3 (87 V)]. (TIF) [file ppat.1002522.s002.tif]

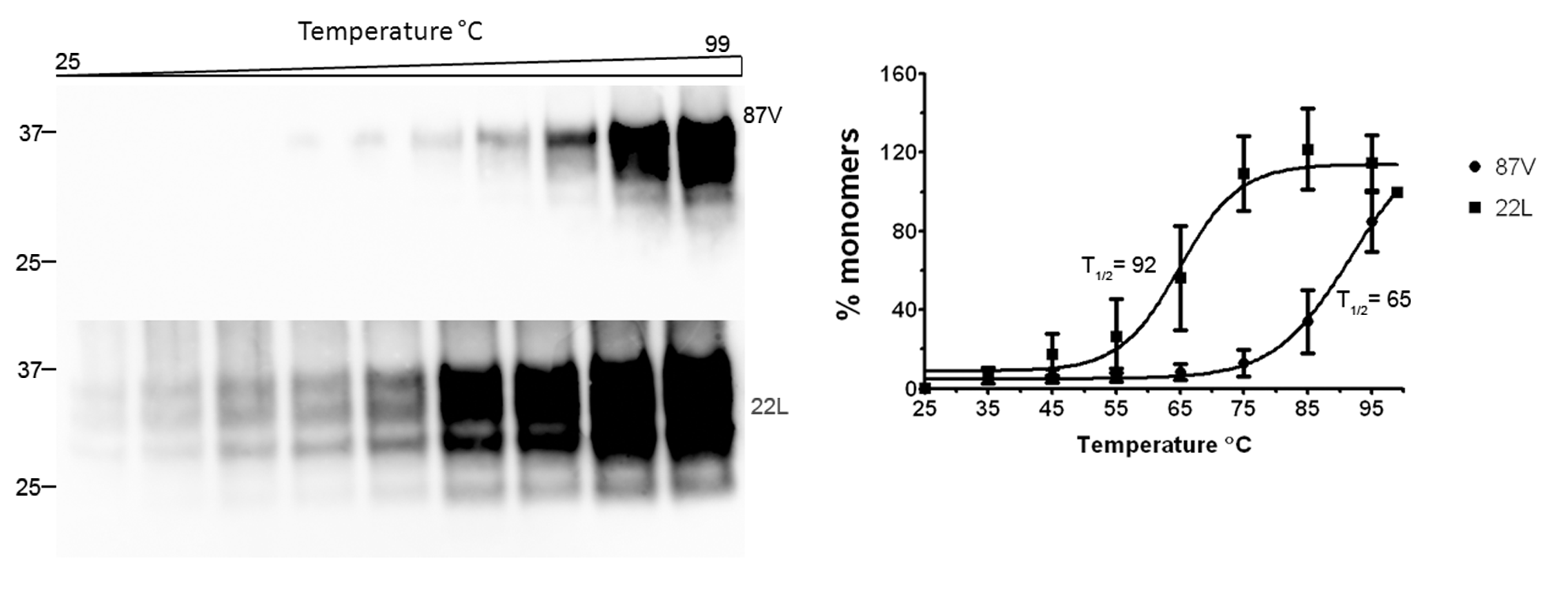

Supplement: Figure S3 — Thermal stability of total insoluble PrPSc for prion strains 22 L and 87 V. (A) The insoluble fraction of brain homogenate was subjected to temperatures from 25°C to 99°C followed by SDS-PAGE. The temperature required for 50% PrPSc disassociation into monomers is lower for the more neuroinvasive prion strain 22 L than for 87 V. (TIF) [file ppat.1002522.s003.tif]
